# Integrated TCM-western “three-team” co-management for type 2 diabetes with sleep disorders: a prospective study

**DOI:** 10.3389/fendo.2026.1918435

**Published:** 2026-08-06

**Authors:** Manfang Sun, Chunyan Li, Yanhua Xia, Hongxiao Yu

**Affiliations:** 1 Department of Endocrinology, Xiamen Haicang Hospital, Xiamen, China; 2 Department of Cardiology, Haicang Hospital, Haicang, Xiamen, China

**Keywords:** integrated Chinese and western medicine, Pittsburgh sleep quality index, prospective study, sleep disorders, type 2 diabetes mellitus

## Abstract

**Background:**

Sleep disorders are common in patients with type 2 diabetes mellitus (T2DM) and are closely associated with poor glycemic control. However, evidence remains limited regarding whether an integrated traditional Chinese and Western medicine “Three-Team” co-management model can improve sleep quality and metabolic outcomes in patients with T2DM and comorbid sleep disorders.

**Objective:**

To evaluate the effects of an integrated traditional Chinese and Western medicine “Three-Team” co-management model on sleep quality, glycemic control, and metabolic parameters in patients with T2DM and comorbid sleep disorders.

**Methods:**

This prospective, concurrent, non-randomized controlled study enrolled 57 patients with T2DM and comorbid sleep disorders who attended the Department of Endocrinology of Xiamen Haicang Hospital between January and December 2025. Patients were assigned to a control group (n = 21) or an intervention group (n = 36) according to patient preference. The control group received conventional glucose-lowering therapy, whereas the intervention group received, in addition to conventional therapy, the integrated Three-Team co-management model involving an endocrinologist, a traditional Chinese medicine physician, and a health manager. The intervention lasted 6 months. Primary outcomes were the Pittsburgh Sleep Quality Index (PSQI), fasting blood glucose (FBG), and glycated hemoglobin (HbA1c). Secondary outcomes included 2-hour C-peptide, total cholesterol (TC), and low-density lipoprotein cholesterol (LDL-C) levels. Longitudinal data were analyzed using mixed-effects linear models.

**Results:**

The intervention group showed significantly greater improvement in PSQI scores over time than the control group (group × time interaction: χ² = 32.59, P < 0.001). At 6 months, the PSQI score decreased by 2.44 points from baseline in the intervention group, representing a 2.4-fold greater reduction than in the control group. Improvements in FBG and HbA1c were also significantly greater in the intervention group (interaction P = 0.047 and P = 0.008, respectively), with significant between-group differences observed as early as 3 months (FBG difference: –2.64 mmol/L, P = 0.018; HbA1c difference: –2.09%, P = 0.002). For secondary outcomes, 2-hour C-peptide increased significantly from baseline in the intervention group (interaction P = 0.015), and reductions in TC and LDL-C were greater than those in the control group (both P < 0.05).

**Conclusion:**

The integrated traditional Chinese and Western medicine “Three-Team” co-management model was associated with earlier and greater improvements in sleep quality and glycemic control in patients with T2DM and comorbid sleep disorders, with additional benefits in postprandial islet function and lipid profile.

## 1 Introduction

China has the largest population of individuals with diabetes worldwide, and type 2 diabetes mellitus (T2DM) represents a major public health burden. Accumulating evidence indicates that sleep disorders are highly prevalent in patients with T2DM, with insomnia affecting approximately 39% of patients and obstructive sleep apnea reported in 55%–86% (1). Studies in Chinese populations have likewise shown a substantial burden of sleep disturbance and abnormal sleep duration among patients with diabetes (2). Importantly, the relationship between sleep disturbance and T2DM appears to be bidirectional. On the one hand, impaired sleep may adversely affect glucose homeostasis through sympathetic nervous system activation, intermittent hypoxia, inflammation, and reduced insulin sensitivity, thereby contributing to poorer glycemic control (3, 4). On the other hand, hyperglycemia, nocturnal glycemic fluctuations, neuropathic pain, and other diabetes-related symptoms may further disrupt sleep continuity and circadian rhythm (5).Despite growing recognition of these interactions, current diabetes management remains largely centered on conventional metabolic targets, such as glycemic and blood pressure control, whereas routine screening and timely intervention for sleep disorders are still insufficient (1). This gap is clinically important because sleep disturbance is associated not only with adverse metabolic outcomes but also with reduced quality of life, greater psychological distress, and poorer diabetes self-management (6).

Xiamen has been a pilot city for hierarchical chronic disease management in China since 2012, and its “Three-Team Co-management” model, involving a specialist physician, a primary care general practitioner, and a health manager, has been progressively implemented in diabetes care (7). Evidence from regional electronic health records showed that this team-based model was associated with a marked shift of diabetes-related visits from general hospitals to community health centers, suggesting improved coordination and delivery of community-based care (7). A subsequent assessment further indicated that the reform was associated with better glucose- and blood pressure-related management indicators at the primary care level, as well as greater acceptance of community-based chronic disease care (8). More broadly, team-based primary care services in China have also been associated with improved patient-perceived quality of care among individuals with non-communicable diseases (9). In parallel, the National Guidelines for the Prevention and Control of Diabetes in Primary Care (2022) incorporated traditional Chinese medicine into the primary-care management framework (10), and later commentary further emphasized an integrated “comanagement of three disciplines” model involving endocrinologists, TCM practitioners, and health management personnel (11). However, published evidence specifically evaluating the effects of this integrated model on sleep quality and comprehensive glycemic outcomes in patients with T2DM and comorbid sleep disorders remains limited. Current evidence in this field has mainly come from targeted sleep-focused interventions, such as general practitioner-delivered cognitive behavioral therapy, rather than integrated TCM–Western medicine co-management models (12, 13).

Building upon the Xiamen “Three-Team Co-management” framework, we incorporated a collaborative traditional Chinese and Western medicine approach. In this model, the endocrinologist is responsible for precision glucose-lowering therapy and complication screening; the TCM physician administers syndrome differentiation-based herbal medicine, acupuncture, and other TCM modalities to holistically manage sleep disturbances; and the health manager provides individualized nutritional and exercise prescriptions along with sleep hygiene guidance. This pathway integrates “quantified glucose control, holistic spirit regulation, and sustained behavioral intervention.” Using the Pittsburgh Sleep Quality Index (PSQI), fasting blood glucose (FBG), and glycated hemoglobin (HbA1c) as primary outcomes, this study systematically evaluated the comprehensive efficacy of this model in patients with T2DM and comorbid sleep disorders, with the aim of providing evidence for integrated traditional Chinese and Western medicine management of psychosomatic comorbidities in diabetes. We hypothesized that the integrated ‘Three-Team’ co-management model would achieve superior improvements in sleep quality and glycemic control compared with conventional therapy alone over the 6-month follow-up period.

## 2 Methods

### 2.1 Study design

This was a prospective, concurrent, non-randomized controlled study. The study protocol was approved by the Ethics Committee of Xiamen Haicang Hospital (approval No. KY-2024033). Written informed consent was obtained from all participants before enrollment.

### 2.2 Study population

A total of 57 patients with type 2 diabetes mellitus (T2DM) and comorbid sleep disorders who attended the Department of Endocrinology of Xiamen Haicang Hospital between January and December 2025 were enrolled.

#### 2.2.1 Diagnostic criteria

T2DM was diagnosed according to the Chinese Guidelines for the Prevention and Treatment of Type 2 Diabetes (2024 edition). Diagnosis was established by any of the following: (1) classic symptoms of diabetes, including polydipsia, polyuria, polyphagia, or unexplained weight loss, with fasting plasma glucose ≥ 7.0 mmol/L; (2) 2-hour plasma glucose ≥ 11.1 mmol/L during an oral glucose tolerance test; or (3) random plasma glucose≥ 11.1 mmol/L. In asymptomatic individuals, repeat confirmatory testing was required. Sleep disorders were diagnosed according to the International Classification of Sleep Disorders and were defined by difficulty initiating sleep, difficulty maintaining sleep, or early-morning awakening accompanied by daytime dysfunction. Poor sleep quality was defined as a Pittsburgh Sleep Quality Index (PSQI) score > 7.

#### 2.2.2 Inclusion criteria

Participants were eligible if they: (1) met the diagnostic criteria for both T2DM and sleep disorders (2); were aged ≥ 18 years; (3) had a PSQI score> 7; (4) were conscious and able to complete questionnaire assessments; and (5) provided written informed consent voluntarily.

#### 2.2.3 Exclusion criteria

Participants were excluded if they: (1) had non-T2DM diabetes, including type 1 diabetes or gestational diabetes; (2) had acute diabetic complications, such as diabetic ketoacidosis or hyperosmolar coma; (3) had severe cardiac, hepatic, or renal insufficiency, or malignancy; (4) had severe infection; (5) had severe psychiatric disorders, such as schizophrenia; (6) were shift workers; or (7) had sleep disturbances attributable to other clearly defined medical conditions, such as chronic pain or pruritus.

#### 2.2.4 Withdrawal criteria

Withdrawal criteria included poor adherence, use of other sleep-improving interventions during the study period, pregnancy, serious adverse events, voluntary withdrawal, or incomplete study data.

### 2.3 Group allocation and interventions

Patients were allocated to the control or intervention group according to their preference.

The control group (n = 21) received guideline-based conventional treatment for type 2 diabetes, including lifestyle management, oral glucose-lowering agents and/or insulin therapy as appropriate, with treatment adjustment according to glycemic status (14, 15). Participants attended outpatient follow-up every 3 months for metabolic assessment, including blood glucose and HbA1c (15).

The intervention group (n = 36) received, in addition to conventional treatment, an integrated traditional Chinese and Western medicine Three-Team Co-management model (16, 17). In this model,three healthcare professionals delivered coordinated, protocol-based interventions. The endocrinologist was responsible for formulating and dynamically adjusting the glucose-lowering regimen based on patients’ glycemic profiles (FBG and HbA1c) at baseline, 3 months, and 6 months. Treatment adjustments followed the Chinese guideline for primary care of type 2 diabetes (15). The TCM physician provided syndrome differentiation-based individualized treatment, including herbal medicine, acupuncture, and acupoint application, administered at each visit (baseline, 3 months, and 6 months). Herbal formulas were tailored to individual patterns (e.g., liver-qi stagnation transforming into fire, heart-spleen deficiency, or yin deficiency with fire excess) and modified at each visit based on symptom changes. Acupuncture points (e.g., HT7, SP6, and PC6) and acupoint applications (e.g., auricular points) were selected according to TCM syndrome differentiation. In addition, patients received instruction in traditional Chinese exercises, including Tai Chi and Baduanjin (17–22), and were encouraged to practice at least 3 times per week, with 30-minute sessions each time. Adherence to exercise was monitored through the health manager’s follow-up records. The health manager delivered structured dietary counseling, exercise guidance, sleep hygiene education, and regular follow-up via an online-offline platform (15, 17). Specifically, the health manager provided individualized meal plans based on patients’ dietary habits and metabolic profiles, along with sleep hygiene recommendations (e.g., regular sleep schedules, avoidance of stimulants, and relaxation techniques). Follow-up contacts were conducted at least every 2 weeks, with additional contacts as needed, to monitor adherence, address barriers, and reinforce behavioral goals. The sleep-management components were informed by previous studies showing that behavioral sleep interventions and community-based non-pharmacological management may improve sleep quality and glycemic outcomes in patients with T2DM and sleep disturbance (23–26).

Both groups were followed for 6 months, and assessments were performed at baseline, 3 months, and 6 months.

### 2.4 Outcome measures

#### 2.4.1 Baseline characteristics

Baseline data included age, sex, height, weight, body mass index (BMI), waist circumference, diabetes duration, and blood pressure.

#### 2.4.2 Primary outcomes

Primary outcomes were sleep quality and glycemic control. Sleep quality was assessed using the Pittsburgh Sleep Quality Index (PSQI), a 19-item self-rated instrument generating seven component scores, including subjective sleep quality, sleep latency, sleep duration, habitual sleep efficiency, sleep disturbances, use of sleep medication, and daytime dysfunction; the global score ranges from 0 to 21, with higher scores indicating poorer sleep quality (27, 28). In the Chinese version, a PSQI score **>** 7 was used to define poor sleep quality (28). PSQI was assessed at baseline and at 3 and 6 months. Glycemic control was evaluated by fasting blood glucose (FBG) and glycated hemoglobin (HbA1c). FBG was measured using a Beckman AU5800 automated biochemical analyzer, and HbA1c was measured using an HA-8180 HbA1c analyzer.

#### 2.4.3 Secondary outcomes

Secondary outcomes included islet function, lipid profile, renal function, liver function, and hemoglobin. Islet function was assessed by fasting C-peptide and 2-hour C-peptide using a Roche Cobas 8000 analyzer.Lipid profile included total cholesterol (TC), triglycerides (TG), high-density lipoprotein cholesterol (HDL-C), and low-density lipoprotein cholesterol (LDL-C), measured using the Beckman AU5800 system. Renal function included serum creatinine, blood urea nitrogen, urinary microalbumin, urinary creatinine, and urinary albumin-to-creatinine ratio (UACR), measured using a MUS9600 automated urine analyzer. Liver function included alanine aminotransferase (ALT) and aspartate aminotransferase (AST). Hemoglobin (Hb) was also recorded.

#### 2.4.4 Specimen collection and processing

Fasting venous blood samples (4 mL) were collected at baseline and at 3 and 6 months. Samples were centrifuged at 3000 rpm for 10 min (centrifugal radius, 13 cm), and the supernatant was used for biochemical analyses.

### 2.5 Statistical analysis

All analyses were performed using Stata version 18.0. Continuous variables were assessed for normality using the Shapiro-Wilk test. Normally distributed variables are presented as mean ± standard deviation (SD) and were compared between groups at baseline using the independent-samples t-test. Non-normally distributed variables are presented as median (interquartile range, IQR) and were compared using the Mann-Whitney U test. Categorical variables are presented as number (percentage) and were compared using the χ² test.

Longitudinal changes in the primary outcomes, including PSQI, FBG, and HbA1c, were analyzed using mixed-effects linear models. Group, time, and the group × time interaction were entered as fixed effects, and subject-specific random intercepts were included to account for within-subject correlation. Parameters were estimated using restricted maximum likelihood (REML). To address potential confounding in this non-randomized design, we adjusted for a comprehensive set of prespecified covariates, including age, sex, BMI, diabetes duration, and baseline values of PSQI, FBG, and HbA1c in all models. When the group × time interaction was significant, *post hoc* comparisons based on marginal means were performed to evaluate between-group differences at each time point and within-group changes from baseline.

To further assess the robustness of the findings against residual confounding, propensity score matching (PSM) was performed using age, sex, BMI, diabetes duration, FBG, HbA1c, and PSQI as matching covariates (see Section 3.6). Sensitivity analyses stratified by median age and analyses excluding extreme values were additionally performed. Sample size considerations: This was an exploratory study; therefore, no formal *a priori* sample size calculation was performed. *Post hoc* power analysis was conducted based on the observed effect sizes for the three primary outcomes at 6 months, using a two-sided α = 0.05. Effect sizes were calculated as Cohen’s d. All tests were two-sided, and P < 0.05 was considered statistically significant. In the mixed-effects models, β coefficients represent the estimated mean differences relative to the control group.

## 3 Results

### 3.1 Participant flow

**Figure 1:**
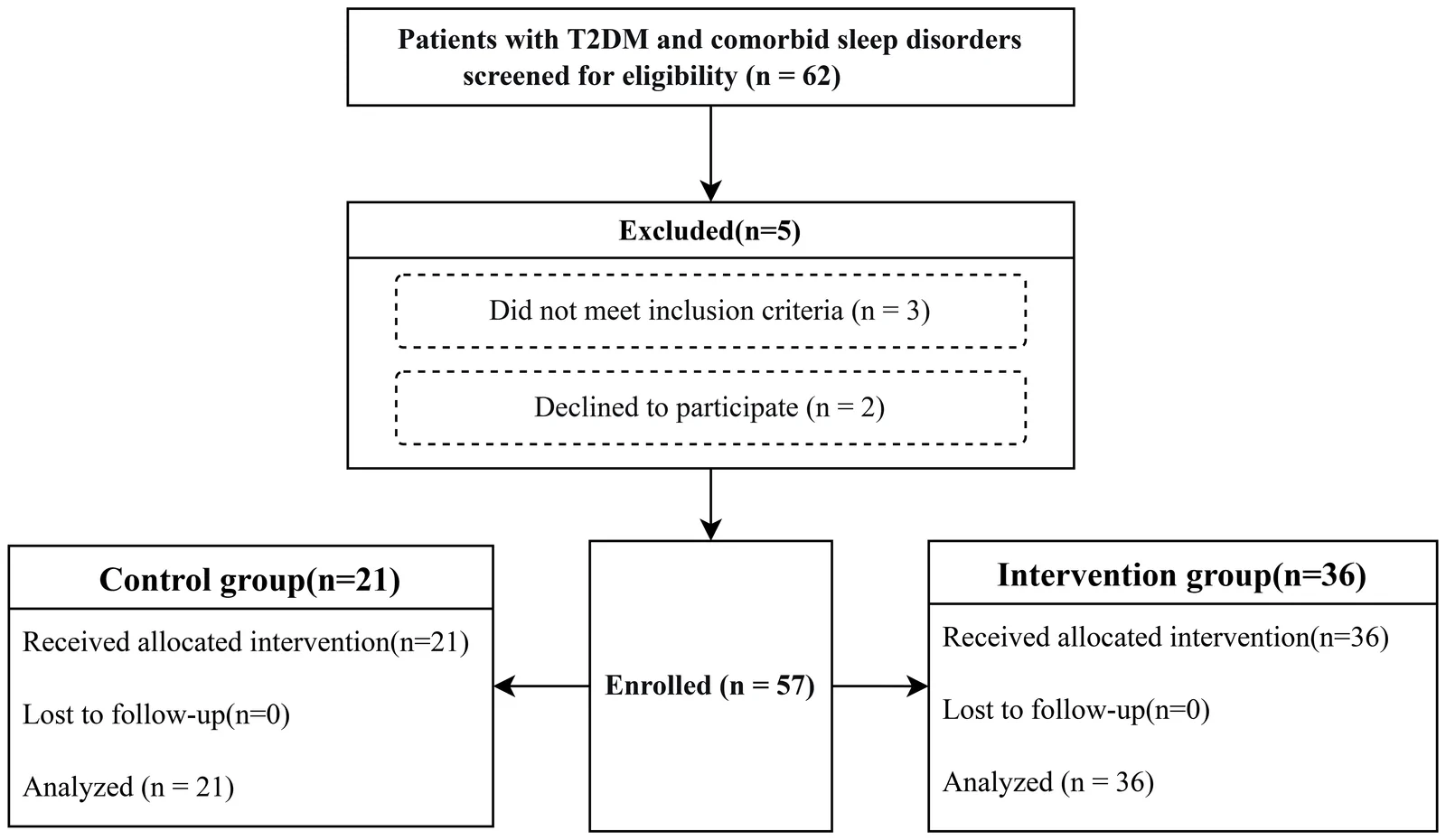
Participant flow diagram. A total of 62 patients with T2DM and comorbid sleep disorders were screened for eligibility. Five patients were excluded (three did not meet the inclusion criteria, two declined to participate). The remaining 57 patients were enrolled and allocated to either the control group (n = 21) or the intervention group (n = 36) according to patient preference. All 57 participants completed the 6-month follow-up without loss to follow-up or discontinuation of the allocated intervention, and all were included in the final analysis. T2DM, type 2 diabetes mellitus.

A total of 62 patients were screened for eligibility. Five patients were excluded (three did not meet the inclusion criteria, two declined to participate). The remaining 57 patients were enrolled and allocated to the control group (n = 21) or intervention group (n = 36) according to patient preference. All 57 participants completed the 6-month follow-up without loss to follow-up or discontinuation of the allocated intervention, and all were included in the final analysis (**Figure 1**).

### 3.2 Baseline characteristics

A total of 57 patients were included in the study, including 21 in the control group and 36 in the intervention group. Baseline demographic, anthropometric, glycemic, sleep-related, hemodynamic, metabolic, hepatic, renal, and islet-function parameters were comparable between groups. No significant between-group differences were observed in age, sex distribution, diabetes duration, BMI, waist circumference, systolic blood pressure, diastolic blood pressure, FBG, HbA1c, PSQI score, TC, TG, HDL-C, LDL-C, fasting C-peptide, 2-h C-peptide, hemoglobin, AST, ALT, blood urea nitrogen, serum creatinine, or UACR (all P > 0.05; **Table 1**).

**Table 1:** Baseline characteristics of the study population.

Characteristic	Control group (n=21)	Intervention group (n=36)	Test statistic	P value
Age (years)	57.10 ± 8.24	56.36 ± 5.23	t = 0.412	0.682
Sex (male/female, n)	11/10	15/21	χ² = 0.614	0.433
Diabetes duration (months)	36 (12, 120)	36 (12, 96)	Z = 0.245	0.807
BMI (kg/m²)	26.13 ± 4.73	24.94 ± 3.59	t = 1.073	0.288
Waist circumference (cm)	94.57 ± 9.40	90.67 ± 9.67	t = 1.486	0.143
SBP (mmHg)	135.57 ± 19.24	130.64 ± 16.61	t = 1.020	0.312
DBP (mmHg)	81.33 ± 8.84	83.69 ± 14.29	t = -0.683	0.497
FBG (mmol/L)	11.86 ± 3.40	11.21 ± 4.02	t = 0.616	0.540
HbA1c (%)	10.92 ± 2.39	10.41 ± 2.27	t = 0.796	0.430
PSQI (score)	13.00 ± 1.64	12.69 ± 1.79	t = 0.641	0.524
TC (mmol/L)	5.50 ± 1.30	5.42 ± 1.70	t = 0.199	0.843
TG (mmol/L)	2.00 (1.44, 3.90)	1.65 (1.03, 2.75)	Z = 0.723	0.470
HDL-C (mmol/L)	1.27 ± 0.33	1.30 ± 0.36	t = -0.267	0.790
LDL-C (mmol/L)	3.47 ± 0.88	3.34 ± 1.18	t = 0.425	0.673
Fasting C-peptide (μg/L)	0.86 ± 0.45	0.68 ± 0.43	t = 1.489	0.142
2-hour C-peptide (μg/L)	1.79 ± 0.85	1.52 ± 1.02	t = 1.034	0.306
Hemoglobin (g/L)	137.86 ± 19.20	137.76 ± 28.80	t = 0.014	0.989
AST (U/L)	29 (23, 52)	22 (18, 40)	Z = 1.291	0.197
ALT (U/L)	31 (22, 83)	22 (15, 47)	Z = 1.497	0.134
Blood urea nitrogen (mmol/L)	5.80 (4.43, 7.67)	5.20 (4.36, 6.28)	Z = 1.531	0.126
Serum creatinine (μmol/L)	61 (56, 77)	65 (57, 70)	Z = 0.090	0.928
UACR (mg/g)	10.29 (5.25, 53.16)	7.96 (4.15, 24.18)	Z = 0.896	0.370

Data are presented as mean ± SD, median (Q1, Q3), or n, as appropriate. For normally distributed continuous variables, between-group comparisons were performed using the independent-samples t-test; for non-normally distributed variables, the Mann-Whitney U test; and for categorical variables, the χ² test. BMI, body mass index; SBP, systolic blood pressure; DBP, diastolic blood pressure; FBG, fasting blood glucose; HbA1c, glycated hemoglobin; PSQI, Pittsburgh Sleep Quality Index; TC, total cholesterol; TG, triglycerides; HDL-C, high-density lipoprotein cholesterol; LDL-C, low-density lipoprotein cholesterol; AST, aspartate aminotransferase; ALT, alanine aminotransferase; UACR, urinary albumin-to-creatinine ratio.

### 3.3 Sleep quality

**Figure 2:**
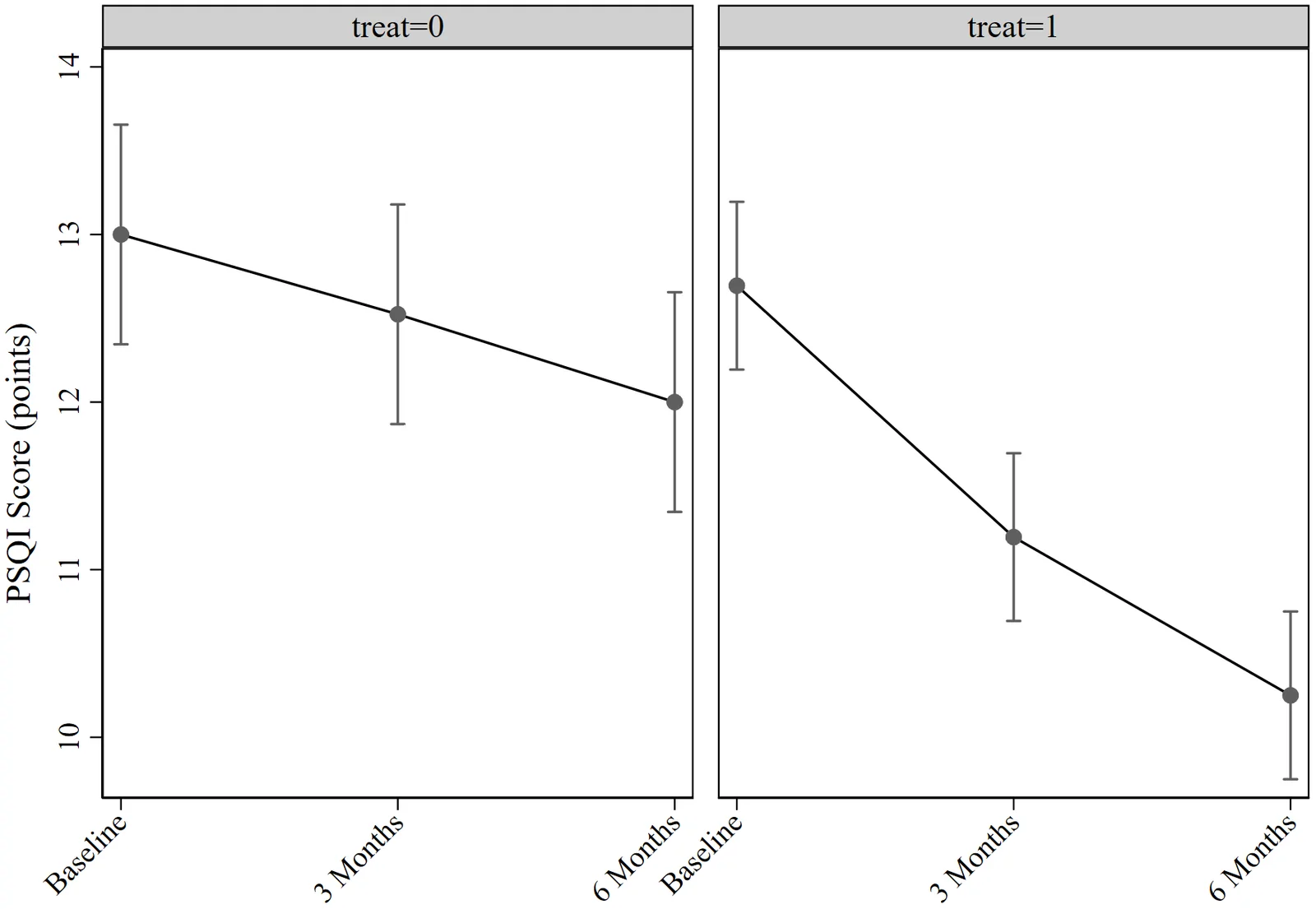
PSQI score changes over time. PSQI, Pittsburgh Sleep Quality Index. Data are presented as marginal mean ± SE estimated from the mixed-effects linear model.

Mixed-effects linear modeling showed a significant group × time interaction for PSQI score (χ²=32.59, P < 0.001), indicating that the pattern of change in sleep quality differed significantly between the two groups (**Table 2**; **Figure 2**). In the intervention group, PSQI score decreased significantly from baseline to 3 months and remained further reduced at 6 months (both P < 0.001,vs. baseline), In the control group, PSQI score also decreased over time, with a modest reduction at 3 months(β=-0.48, P = 0.021) and a greater reduction at 6 months(β=-1.00, P < 0.001). There was no significant between-group difference in PSQI score at baseline(β=-0.31, P = 0.468).However, PSQI scores were significantly lower in the intervention group than in the control group at both 3 months and 6 months, with between-group differences of -1.33 points(P < 0.001) and -1.75 points (P < 0.001), respectively. At 6 months, the reduction in PSQI score from baseline was 2.44 points in the intervention group, which was approximately 2.4-fold greater than that observed in the control group.

**Table 2:** Marginal mean comparisons of PSQI scores between groups.

Time point	Control group (n=21)	Intervention group (n=36)	Difference (intervention – control), points	95% CI	P value
Baseline (0 months)	13.00 ± 0.33	12.69 ± 0.26	-0.31	(-0.89, 0.27)	0.468
3 months	12.52 ± 0.33	11.19 ± 0.26	-1.33	(-1.83, -0.83)	< 0.001
6 months	12.00 ± 0.33	10.25 ± 0.26	-1.75	(-2.25, -1.25)	< 0.001
Interaction effect		χ² = 32.59			P < 0.001

Data are presented as marginal mean ± SE estimated from the mixed-effects linear model. 95% CI, 95% confidence interval for between-group difference. P values represent between-group simple-effect comparisons at each time point.

### 3.4 Fasting blood glucose

**Figure 3:**
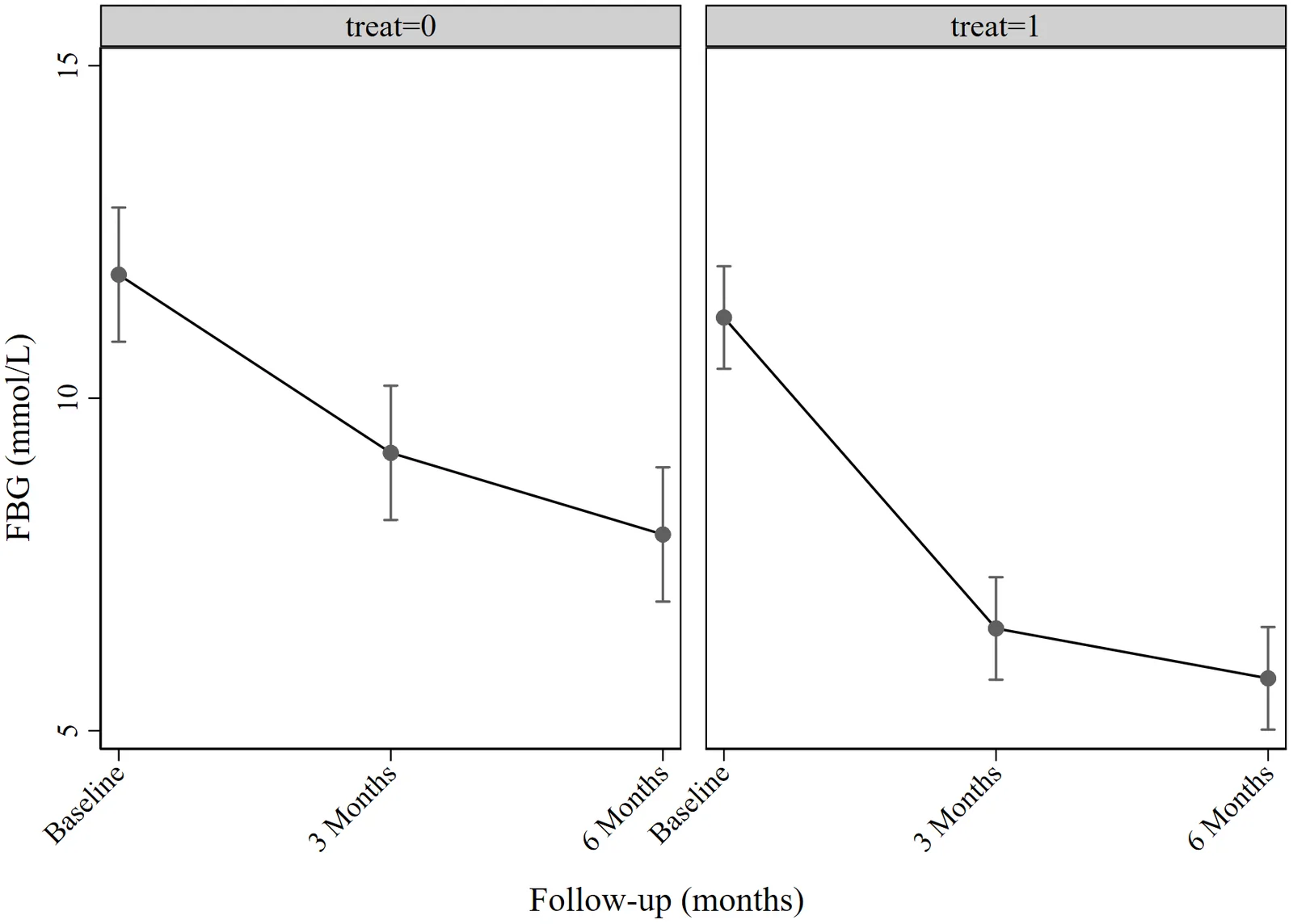
Fasting blood glucose (FBG) changes over time. Data are presented as marginal mean ± SE estimated from the mixed-effects linear model.

Mixed-effects linear modeling showed a significant group×time interaction for FBG(χ²=6.14, P = 0.047), indicating that the pattern of FBG change over time differed between groups (**Table 3**; **Figure 3**).In both groups, FBG decreased significantly from baseline at 3 and 6 months(all P < 0.001,vs. baseline) In the control group, FBG decreased from 11.86 ± 0.51 mmol/L at baseline to 9.18 ± 0.51 mmol/L at 3 months and 7.95 ± 0.51 mmol/L at 6 months. In the intervention group, FBG decreased from 11.21 ± 0.39 mmol/L at baseline to 6.54 ± 0.39 mmol/L at 3 months and 5.79 ± 0.39 mmol/L at 6 months.

**Table 3:** Marginal mean comparisons of FBG between groups.

Time point	Control group (n=21)	Intervention group (n=36)	Difference (intervention – control), mmol/L	95% CI	P value
Baseline (0 months)	11.86 ± 0.51	11.21 ± 0.39	-0.64	(-1.71, 0.43)	0.320
3 months	9.18 ± 0.51	6.54 ± 0.39	-2.64	(-3.87, -1.41)	0.018
6 months	7.95 ± 0.51	5.79 ± 0.39	-2.16	(-3.52, -0.80)	0.071
Interaction effect		χ² = 6.14			P = 0.047

Data are presented as marginal mean ± SE estimated from the mixed-effects linear model. 95% CI, 95% confidence interval for between-group difference. P values represent between-group simple-effect comparisons at each time point.

There was no significant between-group difference in FBG at baseline (β=-0.64, P = 0.320). At 3 months, FBG was significantly lower in the intervention group than in the control group, with a between-group difference of -2.64 mmol/L(P = 0.018). At 6 months, the between-group difference remained numerically evident but did not reach statistical significance (difference: -2.16 mmol/L, P = 0.071).

### 3.5 Glycated hemoglobin

**Figure 4:**
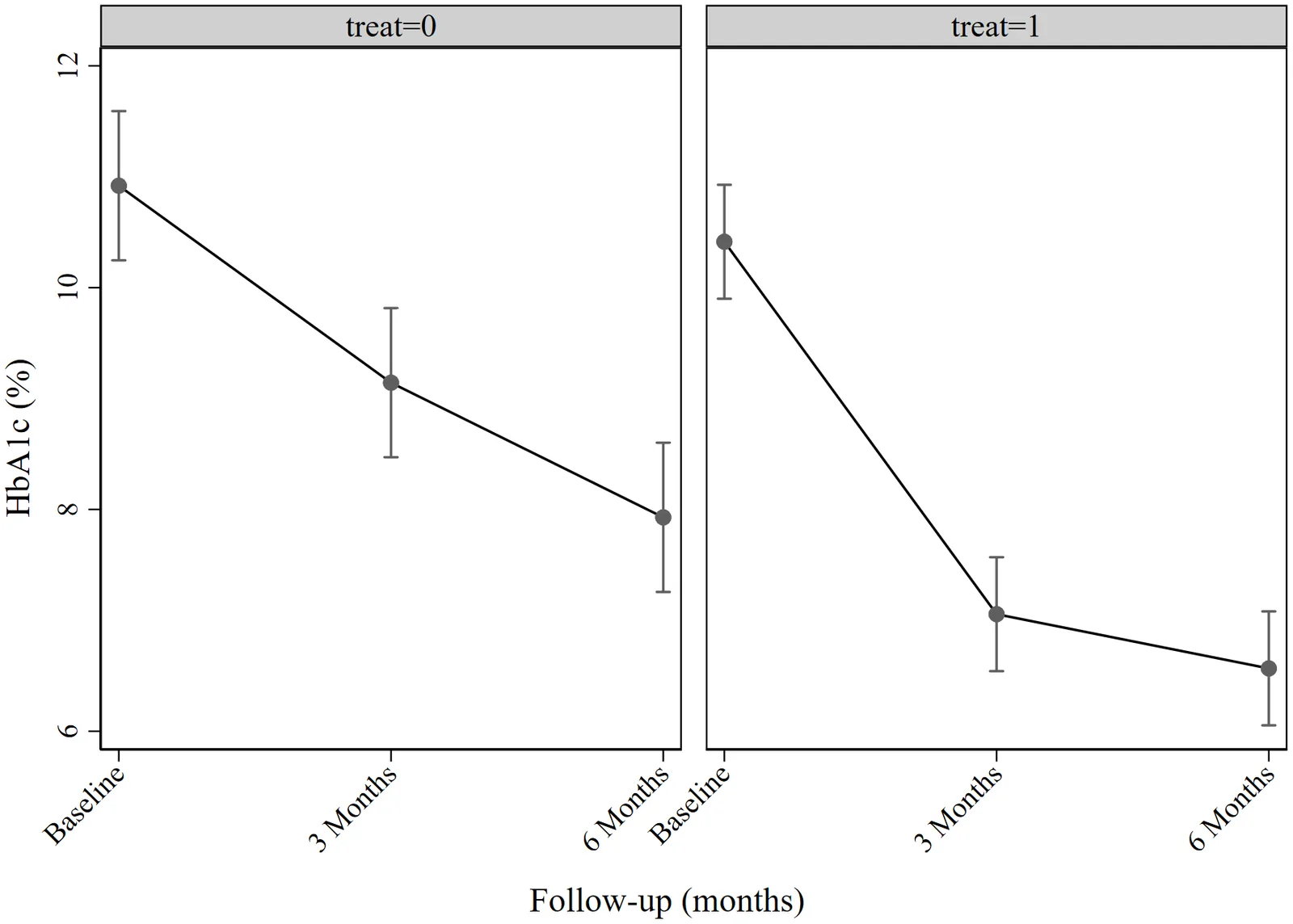
Glycated hemoglobin (HbA1c) changes over time. Data are presented as marginal mean ± SE estimated from the mixed-effects linear model.

Mixed-effects linear modeling showed a significant group × time interaction for HbA1c (χ² = 9.72, P = 0.008), indicating that the temporal pattern of HbA1c change differed between groups (**Table 4**; **Figure 4**). In both groups, HbA1c decreased significantly from baseline to 3 months and 6 months (all P < 0.001, vs. baseline).In the control group, HbA1c decreased from 10.92 ± 0.34% at baseline to 9.14 ± 0.34% at 3 months and 7.93 ± 0.34% at 6 months. In the intervention group, HbA1c decreased from 10.41 ± 0.26% at baseline to 7.06 ± 0.26% at 3 months and 6.57 ± 0.26% at 6 months.There was no significant between-group difference in HbA1c at baseline (β = -0.51, P = 0.242). At 3 months, HbA1c was significantly lower in the intervention group than in the control group, with a between-group difference of -2.09% (P = 0.002).At 6 months, the between-group difference remained numerically lower in the intervention group but did not reach statistical significance(difference: -1.36%, P = 0.092).

**Table 4:** Marginal mean comparisons of HbA1c between groups.

Time point	Control group (n=21)	Intervention group (n=36)	Difference (intervention – control), %	95% CI	P value
Baseline (0 months)	10.92 ± 0.34	10.41 ± 0.26	-0.51	(-1.11, 0.09)	0.242
3 months	9.14 ± 0.34	7.06 ± 0.26	-2.09	(-2.81, -1.37)	0.002
6 months	7.93 ± 0.34	6.57 ± 0.26	-1.36	(-2.11, -0.61)	0.092
Interaction effect		χ² = 9.72			P = 0.008

Data are presented as marginal mean ± SE estimated from the mixed-effects linear model. 95% CI, 95% confidence interval for between-group difference. P values represent between-group simple-effect comparisons at each time point.

### 3.6 Secondary outcome measures

#### 3.6.1 Islet function

A significant group × time interaction was observed for 2-hour C-peptide (χ² = 8.40, P = 0.015). In the intervention group, 2-hour C-peptide increased significantly from baseline to 3 months and 6 months (P = 0.011 and P = 0.005, respectively). In the control group, 2-hour C-peptide showed mild increases over time, but neither reached statistical significance (both P > 0.05). No significant between-group differences were observed at individual time points (both P > 0.05). By contrast, the group × time interaction for fasting C-peptide was not significant (χ² = 2.27, P = 0.321), although both groups showed slight upward trends during follow-up.

#### 3.6.2 Lipid profile

For TC, a significant group × time interaction was observed (χ² = 7.70, P = 0.021). In the intervention group, TC decreased from 5.42 ± 1.70 mmol/L at baseline to 4.00 ± 0.19 mmol/L at 3 months and 3.84 ± 0.19 mmol/L at 6 months (both P < 0.001). In the control group, TC decreased from 5.50 ± 1.30 mmol/L at baseline to 4.71 ± 0.25 mmol/L at 6 months (P = 0.016). Between-group comparisons showed that TC was significantly lower in the intervention group than in the control group at both 3 months (difference: -0.96 mmol/L, P = 0.012) and 6 months (difference: -0.87 mmol/L, P = 0.023).

A significant group × time interaction was also observed for LDL-C (χ² = 6.12, P = 0.047). In the intervention group, LDL-C decreased from 3.34 ± 1.18 mmol/L at baseline to 2.46 ± 0.14 mmol/L at 3 months and 2.39 ± 0.14 mmol/L at 6 months (both P < 0.001). In the control group, LDL-C decreased from 3.47 ± 0.88 mmol/L at baseline to 3.17 ± 0.18 mmol/L at 3 months and 2.91 ± 0.18 mmol/L at 6 months, with a significant reduction observed only at 6 months (P = 0.014). Between-group comparisons showed that LDL-C was significantly lower in the intervention group than in the control group at 3 months (difference: -0.71 mmol/L, P = 0.016), whereas the between-group difference at 6 months was of borderline significance (difference: -0.53 mmol/L, P = 0.096).

No significant group × time interactions were observed for TG or HDL-C (χ² = 0.66, P = 0.718; χ² = 2.23, P = 0.328, respectively), and both groups showed only modest changes during follow-up.

#### 3.6.3 Renal function

Because one patient in the control group had a markedly elevated baseline UACR (3226.18 mg/g), UACR was log-transformed before analysis. After transformation, the group × time interaction for UACR was not significant (χ² = 0.06, P = 0.968). Serum creatinine (χ² = 2.79, P = 0.248) and blood urea nitrogen (χ² = 2.59, P = 0.274) remained stable throughout follow-up, with no evidence of clinically meaningful deterioration.

#### 3.6.4 Other metabolic and anthropometric measures

A significant group × time interaction was observed for ALT (χ² = 6.14, P = 0.046). However, this finding appeared to be largely driven by baseline imbalance, as ALT was higher in the control group than in the intervention group at baseline (61.31 ± 63.82 U/L vs. 39.23 ± 42.63 U/L), whereas values were similar between groups at 6 months (32.41 ± 31.29 U/L vs. 31.41 ± 17.17 U/L, P = 0.876). No significant group × time interactions were observed for BMI, waist circumference, systolic blood pressure, diastolic blood pressure, or AST (all P > 0.05).

### 3.7 Sensitivity analysis

To evaluate the robustness of the primary findings, propensity score matching (PSM) was performed using age, sex, BMI, diabetes duration, FBG, HbA1c, and PSQI as matching covariates. A 1:1 nearest-neighbor matching approach with a caliper of 0.2 was applied. After matching, 52 patients were retained, including 21 in the control group and 31 in the intervention group. Baseline covariate balance after matching was acceptable, with all standardized differences < 10%,and a non-significant overall balance test (P = 0.183). In the matched cohort, the group × time interactions for all three primary outcomes remained statistically significant, including PSQI (χ² = 32.77, P < 0.001), FBG(χ² = 6.18, P = 0.045) and HbA1c (χ² = 11.03, P = 0.004), consistent with the main analysis. Additional sensitivity analyses stratified by median age and analyses excluding extreme values yielded similar results and did not materially alter the main conclusions. *Post hoc* power analysis based on 6-month data showed large effect sizes for all three primary outcomes, with Cohen’s d values of 1.32 for PSQI, 2.72 for FBG, and 1.52 for HbA1c. At a two-sided α = 0.05, statistical power exceeded 99% for all three outcomes.

## 4 Discussion

In the present study, the PSQI was used as the primary outcome to evaluate the efficacy of an integrated traditional Chinese and Western medicine Three-Team Co-management model in patients with T2DM and comorbid sleep disorders. Compared with conventional therapy alone, the intervention group showed earlier and greater improvements in sleep quality, together with more favorable changes in FBG, HbA1c, 2-h C-peptide, and lipid profile. These findings are broadly consistent with previous evidence showing that sleep-focused interventions in T2DM can improve both sleep quality and glycemic outcomes, including community-based cognitive behavioral therapy delivered by general practitioners and structured behavioral sleep interventions (29–31). In addition, recent evidence suggests that non-pharmacological sleep interventions may produce clinically meaningful reductions in HbA1c in patients with T2DM, supporting the view that addressing sleep disturbance can contribute to metabolic improvement (32). Given that sleep disorders in T2DM are highly prevalent and closely linked to adverse metabolic and psychosocial outcomes, our findings further suggest that an integrated collaborative care model may provide added value for the multidimensional management of diabetes with comorbid sleep disturbance (33). This advantage may be attributable to the coordinated integration of three complementary components.

First, the observed benefit may reflect the synergistic effects of pharmacological and non-pharmacological interventions. In addition to conventional glucose-lowering therapy, patients in the intervention group received individualized traditional Chinese medicine (TCM) interventions based on syndrome differentiation, together with acupuncture, auricular acupressure, and traditional exercise therapies. Previous studies have shown that acupuncture-based interventions can improve insomnia-related outcomes, including subjective sleep quality and, in some studies, objective sleep parameters (34, 35). In parallel, structured sleep-focused behavioral interventions in patients with T2DM have been associated with improvements in both sleep quality and glycemic outcomes (36–38). Thus, the integrated strategy used in the present study may have provided additive benefit by combining symptom-oriented sleep management with guideline-based glucose control, rather than relying on a single therapeutic dimension alone.

Second, the findings support the potential value of symptom-driven psychosomatic co-management. Sleep disturbance and T2DM are widely considered to have a bidirectional relationship, in which poor sleep may worsen insulin resistance and glycemic control, whereas diabetes-related symptoms and metabolic dysregulation may further impair sleep (39–41). In the present study, the intervention group showed a significant reduction in PSQI score as early as 3 months, followed by favorable changes in FBG, HbA1c, and lipid parameters. This temporal pattern is consistent with previous evidence suggesting that improving sleep may contribute to subsequent metabolic improvement in T2DM (42–44). Moreover, the increase in 2-h C-peptide observed in the intervention group provides preliminary clinical support for a possible link between sleep improvement and postprandial islet function, although this relationship requires further mechanistic investigation.

Third, sustained behavioral management enabled by coordinated follow-up may also have contributed to the observed effects. Team-based chronic disease management models in China have been associated with improved community-based diabetes care, better care coordination, and higher patient-perceived quality of care (43, 44). In addition, current primary healthcare guidelines for T2DM in China emphasize multidisciplinary management, continuous follow-up, health education, and the use of electronic health records and behavioral interventions in routine care (45). In the present study, the health manager provided ongoing dietary counseling, exercise guidance, sleep hygiene education, and online-offline follow-up, which may have improved treatment adherence and self-management capacity and thereby facilitated the earlier metabolic benefit observed in the intervention group.

Fourth, the intervention was associated not only with better sleep and glycemic control but also with favorable changes in lipid-related outcomes, particularly TC and LDL-C. To our knowledge, few studies have evaluated these domains simultaneously within an integrated TCM-Western medicine co-management framework for T2DM with comorbid sleep disturbance. The lipid-related benefit observed here may reflect the combined effects of improved sleep, intensified lifestyle support, and traditional exercise-based adjunctive management. Previous meta-analyses have shown that traditional Chinese exercises, including Tai Chi and Baduanjin, are associated with improvements in HbA1c, fasting glucose, and lipid parameters in patients with T2DM (45–48). Accordingly, the present findings suggest that the Three-Team Co-management model may have potential value not only for glycemic regulation and sleep improvement but also for broader cardiometabolic risk management.

### 4.1 Limitations

Several limitations should be acknowledged. First, this was a single-center, prospective non-randomized controlled study with a relatively small sample size, which may have introduced selection bias and limits the generalizability of the findings. We acknowledge that allocation by patient preference is subject to confounding, including potential differences in motivation, treatment adherence, and health-seeking behaviors. To mitigate this, we adjusted for multiple prespecified covariates (age, sex, BMI, diabetes duration, and baseline PSQI, FBG, and HbA1c) in the mixed-effects models and additionally performed propensity score matching and sensitivity analyses, all of which yielded consistent results. However, residual confounding cannot be fully excluded, and our findings should be interpreted as effectiveness evidence in a real-world clinical setting rather than proof of causal efficacy. Second, the follow-up period was limited to 6 months, preventing assessment of the long-term durability of the observed improvements in sleep quality and metabolic control, as well as their potential influence on diabetes-related complications. Third, subgroup analyses according to TCM syndrome patterns or specific sleep disorder subtypes were not performed, and therefore the patient subgroups most likely to benefit from this integrated model remain uncertain. Fourth, sleep quality was assessed using the PSQI, which is a subjective measure, and objective sleep assessments such as polysomnography were not available to further validate the findings.

## 5 Conclusions

In conclusion, the integrated traditional Chinese and Western medicine Three-Team Co-management model was associated with improvements in sleep quality, glycemic control, 2-h C-peptide, and lipid-related outcomes in patients with T2DM and comorbid sleep disorders. These findings suggest that a collaborative management strategy integrating specialist-led glucose management, TCM-based symptom regulation, and sustained behavioral support may offer multidimensional benefits in this population. Further multicenter randomized controlled studies with larger sample sizes, longer follow-up, objective sleep assessments, health economic evaluation, and syndrome-stratified analyses are needed to confirm the long-term efficacy, cost-effectiveness, and optimal target population of this model.

## Data Availability

The raw data supporting the conclusions of this article will be made available by the authors, without undue reservation.
